# Supplementary figures and images for: Filling the Void: An Optimized Polymicrobial Interkingdom Biofilm Model for Assessing Novel Antimicrobial Agents in Endodontic Infection
Source: Microorganisms. 2020 Dec 14;8(12):1988. doi: 10.3390/microorganisms8121988 (PMC7764896; doi:10.3390/microorganisms8121988)

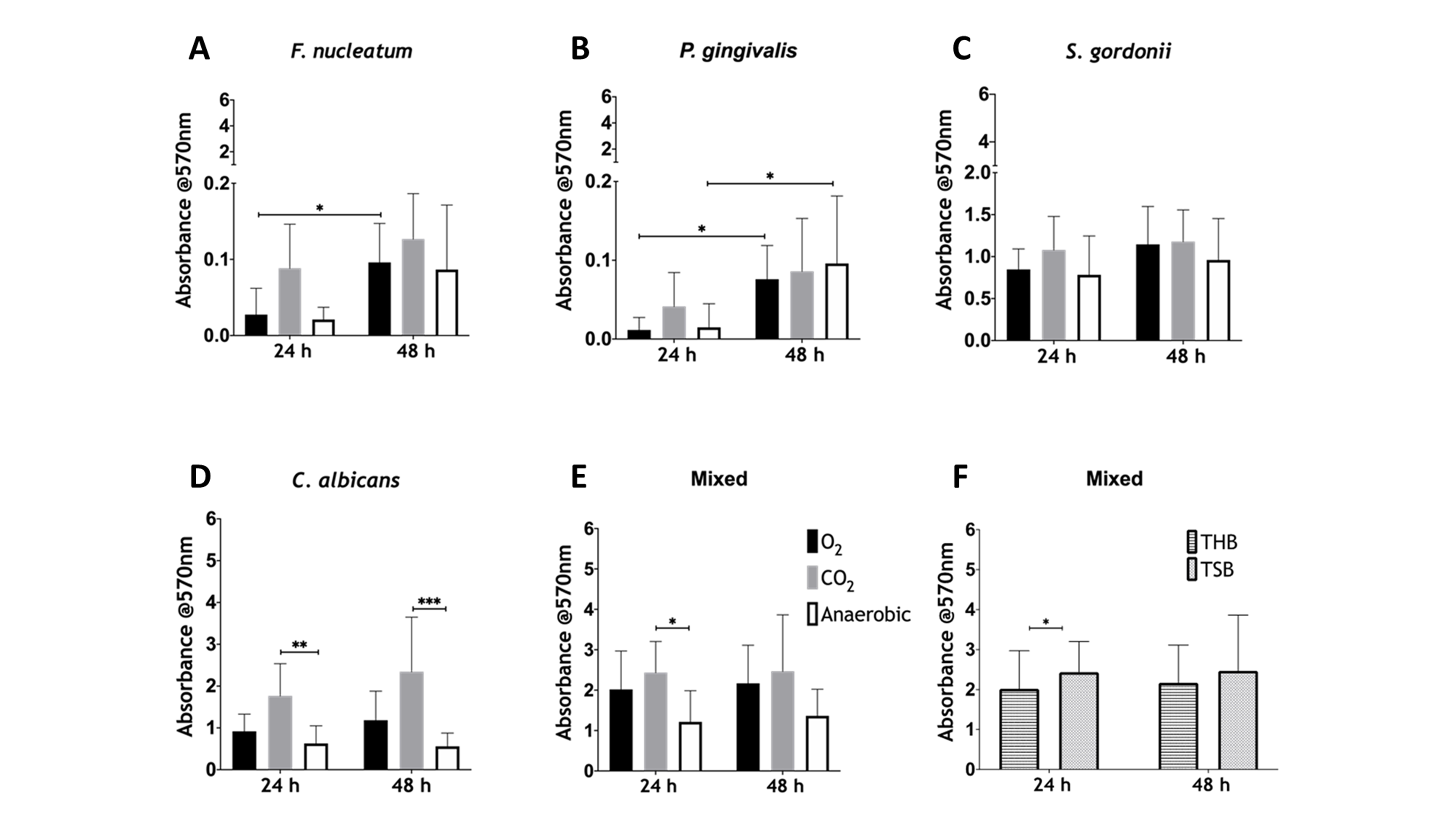

Supplement: Supplementary file 1 [file microorganisms-08-01988-s001.zip › Supplementary/Suppl. Figure 1.png]
